# Trust and digital privacy in healthcare: a cross-sectional descriptive study of trust and attitudes towards uses of electronic health data among the general public in Sweden

**DOI:** 10.1186/s12910-022-00758-z

**Published:** 2022-03-04

**Authors:** Sara Belfrage, Gert Helgesson, Niels Lynøe

**Affiliations:** Stockholm Centre for Healthcare Ethics (CHE), Department of Learning, Informatics, Management and Ethics (LIME), Karolinska Institutet, Tomtebodavägen 18 A, 171 77 Stockholm, Sweden

**Keywords:** Attitudes, Trust, Health data, Research, Sweden

## Abstract

**Background:**

The ability of healthcare to protect sensitive personal data in medical records and registers might influence public trust, which in turn might influence willingness to allow healthcare to use such data. The aim of this study was to examine how the general public’s trust relates to their attitudes towards uses of health data.

**Methods:**

A stratified sample from the general Swedish population received a questionnaire about their willingness to share health data. Respondents were also asked about their trust in the management and protection of electronic health data.

**Results:**

A large majority (81.9%) of respondents revealed high levels of trust in the ability of healthcare to protect electronic patient data. Good health was associated with significantly higher levels of trust compared to bad health. Respondents with low levels of trust were significantly less willing to allow personal data to be used for different purposes and were more inclined to insist on being asked for permission beforehand. Those with low levels of trust also perceived risks of unauthorized access to personal data to be higher and the likely damage of such unauthorized access worse, compared to those with high levels of trust.

**Conclusions:**

Trust in the ability of healthcare to protect electronic health is generally high in Sweden. Those with higher levels of trust are more willing to let their data be used, including without informed consent. It thus seems crucial to promote trust in order to be able to reap the benefits that digitalization makes possible through increased access and use of data in healthcare.

**Supplementary Information:**

The online version contains supplementary material available at 10.1186/s12910-022-00758-z.

## Background

Trust has long been recognized as an important factor for successful interaction in relation to many societal institutions [1, 2]. Its importance has been further stressed in relation to acceptance of expanded access to personal information [3]. Trust is arguably particularly important in the context of healthcare, where people may turn up when they are especially vulnerable and where they need to rely on others on important and personal matters [4]. Since distrust may reduce patients’ willingness to accept others’ access to and use of their personal data, trust has been pointed out as an essential aspect of successful use of electronic health records and other electronically stored health information [5, 6].

Trust in healthcare may refer to trust in healthcare providers and individual medical experts or trust in the healthcare system as a whole [7]. Trust in health care varies between countries [8]. As for variations within countries, a Swedish study indicates that such differences in trust among the general public are primarily associated with micro-level issues, such as interpersonal relationships and the communication skill of healthcare providers—for instance, the patients’ perception of being listened to and taken seriously [9].

Trust in healthcare is usually associated with patient expectations being met in a benevolent manner and patients being offered treatment when needed [4, 5]. Distrust may be a result of previous expectations not being met or with the absence, in the eyes of the patient, of a shared understanding [4, 5]. Trust might colour patients’ perception of interpersonal relationships and outcomes of treatments [10]. High levels of trust might develop into positive feedback loops where trust is continually strengthened; perceptions and attitudes influence behaviour, which in turn affects treatment outcome, which influences perceptions and attitudes, which influence behaviour, and so on [10]. Low levels of trust might work in the opposite way, developing into negative feedback loops [10]. Those who have high levels of trust in healthcare probably seek healthcare when needed, and high levels of trust might also facilitate adherence to treatment. If so, high levels of trust will have beneficial consequences for patient safety. Expected effects for patients with low levels of trust are in many respects the very opposite [10].

The area of e-health and the number of applications of digital solutions in healthcare are growing rapidly, with analysis of data on individuals’ health being a cornerstone. This development gives rise not only to benefits to individuals and society, but also to privacy concerns. Benefits for individual patients include more efficient and safer healthcare, increased possibilities for patient involvement, and more personalised treatments through the use of big data analytics [11–14]. Benefits for the collective, future patients, and society at large include improved possibilities for research on new treatments and organisational methods, quality assurance, identification of suitable treatments, preventive measures for specific groups, and so on [11–14].

Privacy is a broad concept, linked with issues such as secrecy and confidentiality, the protection of data from unauthorized access, and the individual’s own control of personal information [15–17]. The present study concerns the use of electronic health data and the general public’s attitudes relating to privacy. While the relevance of trust has long been stressed in the healthcare context in Sweden as well as internationally [7, 18, 19], there is still a lack of knowledge regarding trust and willingness to share personal health data. How does trust come into play and what are its effects?

The aim of the present study was to examine the association between the general public’s trust in how healthcare manages and protects electronic health data and their attitudes towards different uses of their electronic health data from medical records and health registers. Specific questions of the present study were:

1. Does the general public trust how healthcare manages and protects electronically stored health data?
2. Does the individual’s level of trust in healthcare correlate with attitudes towards use of electronic health data for different purposes, and the disposition to demand informed consent?
3. Does current trust in healthcare correlate with the estimation of the risk of unauthorized access to health data, and the seriousness of such access?
4. What background factors, if any, are particularly associated with trust in healthcare?

## Methods

The present cross-sectional study is based on a questionnaire survey developed and distributed by The Swedish Agency for Health and Care Services Analysis, which also presented overarching results in a report in Swedish [20]. Based on a reanalysis of data from this (first-time) survey, a paper investigating attitudes concerning the use of personal health data has previously been published [11]. The present study is also based on a reanalysis of data, specifically relating to issues of trust.

The study sample consisted of a stratified selection (with 30 strata) of the Swedish population, 18 years old and above (n = 5460). Stratification concerned age and geographical area. Stratification was motivated by the fact that certain age groups tend to respond less frequently than others to questionnaire surveys and the ambition to obtain enough answers for statistical analysis also from members of those groups [20]. The response rate was 30% (n = 1645/5460).

The questionnaire contained 60 questions. Ten of these concerned the respondents’ background and three concerned previous experiences of healthcare, while the rest concerned views and opinions in six areas: the individual’s access to her medical records; access to medical records within the healthcare system; the individual’s control over her medical records; the use of the medical record for other purposes than the individual’s own care; registries and databases where health data is collected; and privacy risks of health data. Three of the questions on views and opinions allowed free-text responses. Out of the ten questions on background, one concerned trust and seven were considered relevant for trust and therefore used in the present paper (see Table 1). The remaining two background questions were not considered useful for analysis in this paper due to how they were phrased.

**Table 1:** Background characteristics of respondents

	High-truster (n = 1283)	Low-truster (n = 286)
In total (n = 1569)	81.9% (80.0–83.8)	18.1% (16.2–20.0)
Sex		
Man (n = 680)	82.1% (79.2–85.0)	17.9% (15.0–20.8)
Woman (n = 884)	81.8% (79.3–84.3)	18.2% (15.7–20.7)
Age		
18–24 years (n = 387)	85.5% (82.0–89.0)	14.5% (11.0–18.0)
25–34 years (n = 344)	83.7% (79.8–87.6)	16.3% (12.4–20.2)
35–49 years (n = 368)	80.4% (76.3–84.5)	19.6% (15.5–23.7)
50–64 years (n = 335)	78.8% (74.5–83.1)	21.2% (16.9–25.5)
65 years and more (n = 121)	77.7% (70.3–85.1)	22.3% (14.9–29.7)
Education		
Primary school (n = 201)	74.6% (68.6–80.6)	25.4% (19.4–31.4)
High school (n = 654)	82.4% (76.4–88.4)	17.6% (11.6–23.6)
University (n = 705)	83.3% (80.5–86.1)	16.7% (13.9–19.5)
Self-reported health status		
Good or very good (n = 1190)	84.2% (82.1–86.3)*	15.8% (13.7–17.9)*
Rather good (n = 303)	75.9% (71.1–80.7)	24.1% (19.3–28.9)
Bad or very bad (n = 72)	66.7% (55.8–77.6)	33.3% (22.4–44.2)
Knowledge about use of medical records		
Good or very good (n = 495)	80.8% (77.3–84.3)	19.2% (15.7–22.7)
Bad or very bad (n = 1059)	82.2% (79.9–84.5)	17.8% (15.5–20.1)
Experience of working within healthcare		
Yes (n = 304)	82.2% (77.9–86.5)	17.8% (13.5–22.1)
No (n = 1260)	81.7% (79.6–83.8)	18.3% (16.2–20.4)
Country of origin		
Sweden or another Nordic country (n = 1406)	81.9% (79.9–83.9)	18.1% (16.1–20.1)
Non-Nordic country (n = 163)	79.1% (72.9–85.3)	20.9% (13.9–27.9)

Background variables of respondents in relation to their trust in how the healthcare system handles and protects patient information from unauthorized access. Very or fairly high trust grouped as ‘high-trusters’, very or fairly low trust grouped as ‘low-trusters’. Results presented as proportions with a 95% confidence interval). A * shows that the CIs are not overlapping (vertically), indicating that if a hypothesis test were conducted the *p*-value would have been < 0.05

In the present study, we focused on issues relevant for trust in healthcare and how trust might influence the respondents’ attitudes and judgments. We examined associations between stated levels of trust in healthcare and respondents’ inclination to allow the use of information from their medical record for quality assurance, medical research, and educational purposes—with or without informed consent. Moreover, we investigated to what degree current levels of trust influenced their estimation of risk of unauthorized access and severity of consequences if electronic health data are exposed to unauthorized access. All questions analysed in this study can be found in Additional file 1: Supplementary materials.

Trust in healthcare was in the questionnaire specified as trust in how healthcare manages and protects electronically stored health data from unauthorized access. Trust in this context refers to current trust and how it might influence judgment and attitudes.

In the analysis, responses stating high or rather high levels of trust in healthcare were collapsed into *high level of trust*. Correspondingly, responses stating low or very low levels of trust were collapsed into *low level of trust*. These two categories were of central interest in the analyses made in the paper. Results are presented as the proportion of respondents with high levels of trust versus respondents with low levels of trust in relation to different background variables (Table 1) and to questions on specific topics, such as accepted data use and informed consent (Tables 2, 3, 4). The results are presented as proportions with a 95% confidence interval; differences between confidence intervals not overlapping each other are considered as significant, comparable with a hypothesis test with a significance level of < 0.05.

Responses such as “I don’t know” and “I don’t have an opinion” were excluded from the analyses. The number of survey respondents providing such responses, together with respondents choosing not to answer the question at all, can be deduced from the tables where the number of respondents of each question is reported.

The study was approved by the research ethical review board in Stockholm (reference number 2018/872-31/5) and was conducted in accordance with relevant legislation and ethics guidelines.

## Results

About four fifths of the respondents, 81.9% (95% CI: 80.0–83.8), stated that they had a very or rather high level of trust in how healthcare manages and protects electronic health data from unauthorized access. Corresponding proportion regarding trust in Swedish authorities in general was 70.6% (95% CI: 68.4–72.8).

Among those who stated that they experienced a good or very good health, 84.2% (95% CI: 82.1-86.3) also stated that they had a very or rather high level of trust in healthcare, compared to those who stated that they experienced a bad or very bad health, where only 66.7% (95% CI: 55.8-77.6) also stated that they had a very or rather high level of trust in healthcare. Respondents’ perception of their own health was the only background factor of those we analysed (sex, age, education, health, self-estimated knowledge of the use of medical records within healthcare, and own experience from working in healthcare) that made a significant difference between those with high and low levels of trust (Table 1).

### Trust and the use of electronic health data with and without informed consent

A majority of the respondents stated that they were prepared to allow the use of digital information from medical records and health registers for quality assurance of healthcare, for research purposes, and for clinical education under certain conditions. The level of trust in healthcare was associated with respondents’ willingness to allow these uses with and without informed consent. Significantly more of those with high levels of trust accepted the use of health data without informed consent compared to those with low levels of trust. The proportion requesting informed consent was higher for both high-level and low-level trusters when asked about use for medical research and even more so for clinical education, compared to use for quality assurance (Table 2).

**Table 2:** Attitudes to different uses of electronic health data with or without consent

	No never	Yes with IC*	Yes without IC*
Medical follow up and of the quality of healthcare			
High-truster (n = 1177)	2.1% (1.3–2.9)	47.2% (44.3–50.1)	50.7% (47.8–53.6)
Low-truster (n = 243)	5.8% (2.9–8.7)	66.2% (60.3–72.1)	28.0% (22.4–33.6)
Research			
High-truster (n = 1173)	2.3% (1.4–3.2)	59.4% (56.6–62.2)	38.3% (35.5–41.1)
Low-truster (n = 243)	7.0% (3.8–10.2)	68.7% (62.9–74.5)	24.3% (18.9–29.7)
Clinical education			
High-truster (n = 1142)	3.3% (2.3–4.3)	66.0% (63.3–68.7)	30.7% (28.0–33.4)
Low-truster (n = 229)	10.0% (6.1–13.9)	69.9% (64.0–75.8)	20.1% (14.9–25.3)

The attitudes of those with high levels and low levels of trust (‘high-trusters’ and ‘low-trusters’), respectively, towards allowing authorized staff to use information in medical records for quality assurance, research, and educational purposes. Results presented as proportions with a 95% confidence interval

*IC informed consent

### Trust and estimation of risks and severity of consequences

The relations between respondents’ trust in healthcare and their estimations of (a) the risk of unauthorized access when healthcare units share medical records and (b) the severity of the consequences if their medical records were to be exposed to unauthorized access were also examined. Those with high levels of trust estimated the risks as significantly lower [26.6% (95% CI: 23.8–29.4)] than did those with low levels of trust [59.3% (95% CI: 52.6–66.0)]. Those with high levels of trust also thought consequences would be serious if unauthorized persons accessed their medical records [30.3% (95% CI: 27.4–33.2)] to a significantly lower degree than those with low levels of trust [54.0% (95% CI: 47.3–60.7)] (Table 3).

**Table 3:** Perceptions of risks and consequences of unauthorized reading of medical records

	I think the risk of unauthorized persons reading information from my record would be high	I think the consequences for me if unauthorized persons read my data would be serious
High-truster (n = 959/980)	26.6% (23.8–29.4)	30.3% (27.4–33.2)
Low-truster (n = 209/211)	59.3% (52.6–66.0)	54.0% (47.3–60.7)

Estimations by individuals with high levels of trust (here ‘high-trusters’) and individuals with low levels of trust (here ‘low-trusters’) regarding the risk of unauthorized access to medical records if different healthcare units were to share medical records, and valuations of the consequences of such access. Results presented as proportions of those who responded “completely correct” or “fairly correct” with a 95% confidence interval

The estimation of how serious different kinds of unauthorized access would be was also investigated in relation to trust. A difference between those with high and those with low levels of trust was identified. This difference varied depending on the nature of the unauthorized access. Staff reading information from a patient’s medical record without having a professional need to do so was considered a more serious action by those with low compared to those with high levels of trust. The more serious the action was considered to be by all respondents, the lesser the difference in estimation of seriousness between the groups. For instance, there was no significant difference among the two groups if the action concerned hackers who got access to electronically stored sensitive personal information and spread it (Table 4).

**Table 4:** Perceived seriousness of different actors accessing electronic health data

	Trust	Very serious
Healthcare staff involved in my care but who do not need access to that data	High (n = 1232) Low (n = 269)	32.8% (30.2–35.4) 45.4% (39.4–51.4)
Healthcare staff not involved in my care but who know me socially	High (n = 1245) Low (n = 280)	50.8% (48.0–53.6) 68.1% (62.6–73.6)
Employers/insurance companies/banks who gain access to the data and can evaluate me based on them	High (n = 1235) Low (n = 269)	77.1% (74.8–79.4) 86.2% (82.1–90.3)
So called hackers who gain access to the medical record system and pass data forward	High (n = 1247) Low (n = 268)	88.7% (86.9–90.5) 91.8% (88.5–95.1)

Estimations by individuals with high levels of trust and individuals with low levels of trust regarding how serious it would be if different actors got access to their data in medical records or registers. Results presented as proportions of those who responded “very serious” with a 95% confidence interval

## Discussion

### The level of trust is high and seems to influence views on health data use

The results of this study suggest that there is considerable trust in Swedish healthcare and its ability to protect electronic health data among the respondents. Prior results show that the Swedish population has a significantly higher level of trust in healthcare as well as in other public authorities compared to other European countries and to the U.S. [8, 21, 22]. However, the results of this study also show that the respondents’ attitudes towards the use of their health data varied with their level of trust in healthcare. Those with low levels of trust were less willing to allow personal data to be used for different purposes and were more inclined to insist on being asked for permission beforehand. Those with low levels of trust also perceived risks of unauthorized access to personal data to be higher and the likely damage of such unauthorized access worse, compared to those with high levels of trust. Our interpretation is that trust influences attitudes and judgments in these respects.

Among both those with high and those with low levels of trust, rather few respondents rejected use of medical records for quality assurance, medical research, or clinical education purposes, i.e., for uses beyond their own immediate health interests. However, among those with high levels of trust, the proportion of respondents rejecting such uses was lower than among those with low levels of trust. There was a tendency among both those with high and those with low levels of trust to perceive quality assurance as a more acceptable use than medical research and even more so than clinical education purposes, even though the latter group was less positive than the former towards all uses. Quality assurance might perhaps be understood as more directly linked to the quality of present patients’ care, whereas research might be understood as generating less immediate benefits. Although it should be obvious that clinical education is a precondition for the existence of professional healthcare and high-quality treatment, use of data for educational purposes was treated with greater caution by the respondents.

Also a majority of those with high levels of trust regarded informed consent as a precondition for accepting that their medical record be used for medical research or clinical education. Nevertheless, trust seems to have influenced the responses, since those with low levels of trust to a greater extent considered informed consent a precondition for their approval. This is in accordance with previous studies [23, 24].

If our analysis is correct, it is imperative to maintain and increase the level of trust among the public, as this seems to be a precondition for broad acceptance of the use of health data for purposes of important social value beyond the benefits of the individual patient. Mechanisms of trust therefore warrant further study [18].

### Levels of trust associated with self-reported health

A surprising result was that self-reported good health was associated with high levels of trust while self-reported bad health was associated with lower levels of trust. One explanation of this result could be that those with good health have fewer contacts with healthcare and therefore fewer potential occasions where they might get disappointed, while those in poor health have more contacts with healthcare and to a greater extent lose trust due to experienced realities. However, the result disagrees with some prior findings for which the opposite pattern of explanation was offered in [23]. Based on our result, we suggest that one’s trust in how healthcare handles electronic health data is influenced by one’s general trust in healthcare. It is known from previous studies that patients with negative healthcare encounters mistrust healthcare to a greater extent [9]. Another possible interpretation, in line with the feedback loops described in the introduction, is that those with limited trust tend to underestimate their own health status, while those with higher trust tend to do the opposite.

The connection between levels of trust, feeling wronged, and levels of health needs to be further explored.

### Estimations of risks influenced by trust

The results of this study further indicate that trust in healthcare is associated with estimations of risks of unauthorized access to health data, and the severity of consequences following of such unauthorized access. Those with high levels of trust tend to estimate risks as lower and subsequent consequences as less serious, compared to those with low levels of trust. Relations between levels of trust and estimations of risk have been reported previously [18].

In other words, trust seems to influence the perception of reality. One way to interpret this is that one learns through previous experience what to expect (correct or not) about risks and consequences if things go bad. This is not to deny that trust may also be influenced by other factors, for instance negative reports in traditional and social media, but how such news are perceived may be strongly influenced by what level of trust one already has [23, 25, 26].

### Implications

To be worthy of trust, one must be reliable in fulfilling expectations. However, to be trusted, it is not enough that one consistently acts in a trustworthy way and has the will to do so—the person trusting will also have to perceive that this is the case [27]. In the context of the present paper, patients’ trust in the healthcare system arguably has to do with the actual and perceived trustworthiness of its handling and protection of patient information from unauthorized access.

What healthcare can do to prove its competence and willingness to act in a way worthy of trust [27] is to set up and follow proper reliable routines for handling sensitive personal data—and to communicate with the individuals concerned that appropriate steps are taken and that the matter is taken seriously. This means that maintenance of trust requires reliable systems of data storage and retrieval, but also that the provider–patient communication is satisfactory both regarding the patient’s needs and regarding how data is protected. The communication part might involve taking time to listen to patients on how they prefer their data to be used and explain the ways in which their data will handled, including who will have access. It might also involve reassuring them of the safeguards in place to protect their privacy and ensuring they are aware of existing possibilities to influence how their data are to be used.

With reliable and well communicated routines in place, patients are more likely to end up in a positive feedback loop regarding trust, which facilitates use of electronic health records and other electronically stored health information in ways promotive to health, which in turn provides further appreciation of the reliability and productiveness of the system. Again, it is important not only that patients trust the handling of personal data, but also that this trust is adequate and deserved [28]. If not, short term benefits from data sharing may be turned into harms to patients who experience their privacy disrespected and as a consequence become less willing to share data and possibly also less inclined to be truthful in their encounters with healthcare.

### Strengths and limitations

The low response rate of the questionnaire (30%) reflects a trend during the last decades of decreased response rates in survey-based research carried out in Sweden [20]. Well-known recurrent surveys also face this challenge [29]. This particular survey was made for the first time, was cognitively demanding, and required proficiency in Swedish. Furthermore, the government agency distributing it was formed only five years earlier and is probably not that well known. All these are factors that might be relevant in explaining the low response rate. The stratification procedure applied in the survey, inviting more potential participants from groups who usually have low response rates in questionnaires, probably further lowered the average response rate.

However, even though the response rate is quite low, it is not obvious that the main results—that levels of trust in healthcare influenced participants’ estimations and attitudes towards various issues associated with the use of health data—would have been different if the response rate had been higher. As in all cross-sectional studies, there is a risk for selection bias, particularly if the response rate is low.

Previous research indicates that the general public primarily associates trust in healthcare with interpersonal relationships (9). This could be a problem for the present study since trust in healthcare here refers to trust in the ability of healthcare to manage and protect electronic health data from unauthorized access. However, it may not, since previous research could be understood as saying merely that trust levels are influenced by micro-level events.

## Conclusions

Trust in the ability of healthcare to manage and protect sensitive health information in medical records and health registers from unauthorized access is high among the respondents in this study, as is the willingness to allow health data to be used for purposes with no immediate benefit to the individual patient. Our results suggest that estimations of risk of data breaches, and the severity of consequences if such events were to take place, are influenced by respondents’ level of trust, as is their attitudes towards the need to apply informed consent procedures for use of patient data.

Our results show that trust is crucial for broad acceptance of uses of health data for a variety of socially valuable purposes. To protect and promote trust is therefore of outmost importance for reaping the benefits of digitalization in the healthcare setting.

## Supplementary Information

**Additional file 1**. Supplementary materials.

## Data Availability

The data that support the findings of this study are not openly available due to reasons of sensitivity and are available from the corresponding author upon reasonable request. Data are located in controlled access data storage at Karolinska Institutet.
