# Supplementary material for: Trust and digital privacy in healthcare: a cross-sectional descriptive study of trust and attitudes towards uses of electronic health data among the general public in Sweden
Source: BMC Med Ethics. 2022 Mar 4;23:19. doi: 10.1186/s12910-022-00758-z (PMC8896318; doi:10.1186/s12910-022-00758-z)
Supplement: Supplementary file 1 — Additional file 1. Supplementary materials. [file 12910_2022_758_MOESM1_ESM.docx]

## Supplementary material: Survey questions used in this study

1. What do you think about the following statements?

Generally, authorized staff should be allowed to use information in medical records for:

1. Medical follow up of the quality of healthcare?
2. Certain research?
3. Education within healthcare?

- No, never
- Yes, but only with patient consent
- Yes, even without patient consent
- I don’t know/I don’t have an opinion

1. What is your view on different care units getting access to each other’s medical records?

- I think the risk of unauthorized persons reading information from my record would be high
- I think the consequences for me if unauthorized persons read my data would be serious

Completely correct Fairly correct Fairly incorrect Completely incorrect

1. How serious would you consider it to be if any of the following actors got access to your data in medical records or registers?

- Healthcare staff not involved in my care but who knows me socially
- Healthcare staff involved in my care but who do not need access to that data
- So called hackers who gain access to the medical record system and pass data forward
- Employers/insurance companies/banks who gain access to the data and can evaluate me based on them

Not very serious Rather serious Very serious

1. On the whole, how much trust do you have in...

...Swedish authorities in general

...how the healthcare system handles and protects patient information from unauthorized access

Very high trust Fairly high trust Fairly low trust Very low trust

1. Are you…?

- Man
- Woman
- That distinction does not fit me

1. Which year were you born? _ _ _ _
2. What is your highest level of education?

- Primary school or equivalent
- Secondary school or equivalent
- University education or equivalent

1. How do you estimate your general health?

- Very good
- Good
- Fair
- Bad
- Very bad

1. Where were you born?

- Sweden or another Nordic country
- Another European country
- Country outside of Europe

1. Do you work, or have you previously worked, within healthcare in contact with patients?

- Yes
- No

1. How would you rate your knowledge about…

… how medical records are used within healthcare today?

Very good Fairly good Fairly poor Very poor
